# High salt diet does not impact the development of acute myeloid leukemia in mice

**DOI:** 10.1007/s00262-022-03244-y

**Published:** 2022-07-08

**Authors:** Mathangi Janakiraman, Natallia Salei, Gurumoorthy Krishnamoorthy

**Affiliations:** Research Group Neuroinflammation and Mucosal Immunology, Max Planck Institute of Biochemistry, Martinsried, Germany

**Keywords:** Microbiota, Acute myeloid leukemia, High salt diet, Tumor immunology

## Abstract

The gut microbiota has not only been implicated in the development of some cancers but has also been shown to modulate the efficacy of cancer therapeutics. Although the microbiota is an attractive target in cancer therapy, there is limited data available regarding the relevance of microbiota and dietary interventions in the various types of tumors. Recently, a high salt diet (HSD) has attracted attention in cancer development owing to its profound effects on modulating microbiota and immune responses. Here, we investigated the impact of HSD on microbiota, immune responses, and the development of acute myeloid leukemia using two syngeneic transplantation models. HSD significantly changes the microbiota composition, TH17 responses, and NK cells. However, we found no influence of HSD on tumor development. The kinetics and characteristics of tumor development were similar despite varying the number of injected tumor cells. Our data show that the effects of the microbiome and dietary interventions can be tumor-specific and may not apply to all types of cancers.

**Supplementary Information:**

The online version contains supplementary material available at 10.1007/s00262-022-03244-y.

## Introduction

Acute myeloid leukemia (AML) is a common life-threatening form of leukemia in adults which is caused by the uncontrolled proliferation of myeloid cells that are blocked at different stages of maturation [1]. Several studies have identified various immunosuppressive mechanisms in AML like the induction of T cell and NK cell dysfunction, and expansion of T_reg_ cells and myeloid-derived suppressor cells (MDSCs) [2]. Recently, several studies have shown that the gut microbiome can impact anti-tumor immune responses as well as treatment-induced immune responses in certain types of cancers [3]. However, the precise link between the gut microbiota, systemic immune alterations, and tumor immune evasion in different types of cancers is still to be fully elucidated. While several studies have associated gut microbial dysbiosis with the genesis of acute leukemia [4–6], currently there is insufficient data to implicate specific gut microbiota or specific dietary components to the treatment of AML.

Dietary components play an essential role in human health by regulating the gut microbiota, cellular metabolism, and immune homeostasis. Excessive intake of dietary salt that is commonly found in processed foods has been correlated with a high risk of cardiovascular disease and autoimmune diseases [7]. Indeed, growing evidence suggests that salt consumption causes immune dysregulation by affecting innate and adaptive immune responses [7–10]. A high salt diet (HSD) has also been shown to enhance anti-tumor immune responses in mouse models of melanoma and mammary cancer through changes in gut microbiota and the MDSC and NK cells populations [10–12]. HSD has also been reported to promote breast cancer progression and epidemiological studies show an association between HSD and increased risk for gastric cancers [13–15]. These results are consistent with the observations that HSD feeding facilitates the accumulation of sodium in the skin and the gut, thus enhancing barrier immune responses [16]. Further, HSD is also known to affect systemic immune responses albeit at reduced levels compared to the barrier immune responses. It is currently unclear as to whether HSD would also affect the development of other tumor types, especially hematologic cancers. In the present study, we investigated the impact of HSD on cancer development in mouse models of AML.

## Results

### A high salt diet (HSD) is well tolerated by mice

First, to identify systemic alterations induced by HSD that might be relevant in anti-tumor responses, we fed young adult wild type (WT) C57BL/6 mice with control food pellets (C1000) or food pellets with 4% NaCl (HSD) for 3–4 weeks. These two food pellets were similar in their composition except for NaCl (Supplementary Fig. 1a). Since increased salt feeding is associated with cardiovascular complications [17], we carefully monitored HSD-fed mice for various physiological changes. We observed no major changes in their food intake. Bodyweight and serum leptin levels were similar in control and HSD-fed animals indicating the general tolerability of this diet regimen without any caloric restriction (Supplementary Fig. 1b, c). We also performed a complete blood count analysis, and while we observed changes in the hemoglobin concentrations and marginal differences in the monocyte frequencies under C1000 and HSD, there were also no gross changes in their other blood values (Supplementary Fig. 1d).

### Gut microbiota alterations induced by HSD

**Fig. 1:**
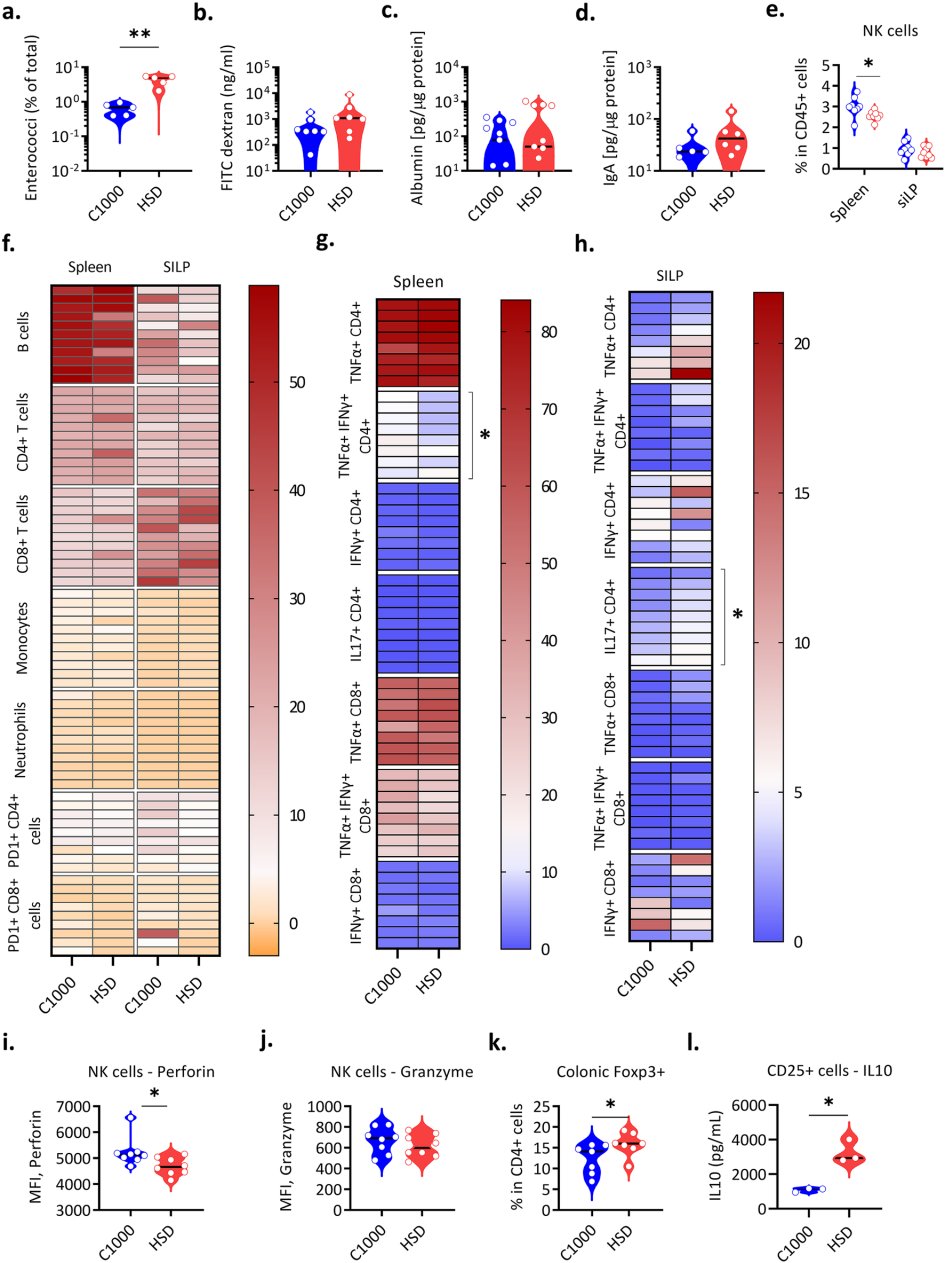
HSD-induced alterations in the intestine and immune cell populations. (**a**–**l**) WT C57BL/6 mice were weaned onto C1000 or HSD and fecal content, sera, and organs were collected 3–4 weeks after the diet switch. **a** 16 s rRNA qPCR analysis of fecal DNA with Enterococci specific primers represented as a percentage of total microbiota (C1000 *n* = 5, HSD *n* = 5). **P = 0.0079 (Mann Whitney’s *U* test). Each circle represents an individual mouse. **b** FITC-Dextran levels in the sera of C57BL/6 mice fed C1000 (*n* = 7) and HSD (*n* = 7). 3–4 weeks after the diet switch, mice were gavaged FITC-dextran and sera was collected after 4 h. Each circle represents an individual mouse. **c** Fecal albumin levels in WT C57BL/6 mice fed C1000 (*n* = 8) and HSD (*n* = 8), quantified by ELISA and normalized to total protein levels. Each circle represents an individual mouse. **d** Fecal IgA levels in C57BL/6 mice fed C1000 (*n* = 5) and HSD (*n* = 6), quantified by ELISA and normalized to total protein levels. Each circle represents an individual mouse. **e** The frequencies of NK cells (expressed as % of CD45^+^ cells), were obtained by flow cytometry analysis in the spleen and small intestine lamina propria (siLP) of C57BL/6 mice fed with C1000 (*n* = 8) and HSD (*n* = 8). *P = 0.0206 (Mann Whitney’s *U* test). Each circle represents an individual mouse. Data from 2 individual experiments are pooled. **f** Heatmap showing the frequencies (% of CD45^+^ cells) of various immune cell subsets obtained by flow cytometry analysis in the spleen and siLP of C57BL/6 mice fed with C1000 (*n* = 9–11) and HSD (*n* = 9–11). Data from 3 individual experiments are pooled. **g** Heatmap showing the frequencies (% of CD4^+^ or CD8^+^ T cells) of various cytokine-producing T cell subsets obtained by flow cytometry analysis in the spleen of C57BL/6 mice fed with C1000 (*n* = 8–9) and HSD (*n* = 8–9). *P = 0.0351 (Mann Whitney’s *U* test). Data from 2 individual experiments are pooled. **h** Heatmap showing the frequencies (% of CD4^+^ or CD8^+^ T cells) of various cytokine-producing T cell subsets obtained by flow cytometry analysis in the siLP of C57BL/6 mice fed with C1000 (*n* = 8–9) and HSD (*n* = 8–9). *P = 0.0315 (Mann Whitney’s *U* test). Data from 2 individual experiments are pooled. **i** The mean fluorescence intensity (MFI) of Perforins in splenic NK cells, obtained by flow cytometry analysis of C57BL/6 mice fed with C1000 (*n* = 8) and HSD (*n* = 8). *P = 0.0147 (Mann Whitney’s *U* test). Each circle represents an individual mouse. Data from 2 individual experiments are pooled. **j** The mean fluorescence intensity of Granzyme in splenic NK cells, obtained by flow cytometry analysis in C57BL/6 mice fed with C1000 (*n* = 8) and HSD (*n* = 8). Each circle represents an individual mouse. Data from 2 individual experiments are pooled. **k** The frequencies of Foxp3^+^ cells in the colonic lamina propria (% of CD4^+^ T cells) of WT C57BL/6 mice fed C1000 (*n* = 7) and HSD (*n* = 7), obtained by flow cytometry analysis. *P = 0.0291 (Mann Whitney’s *U* test). Each circle represents an individual mouse. Data from 2 individual experiments are pooled. **l** IL-10 levels in lymph node T_reg_ cells isolated from C57BL/6 mice 3–4 weeks after diet switch and cultured in the presence of anti-CD3 and anti-CD28. CD4^+^ CD25^+^ cells were sorted from the lymph nodes of C1000 (*n* = 3) and HSD (*n* = 3) -fed mice. *P = 0.0270 (*T*-test with Welch’s correction). Each circle represents a pool of 2 mice. One representative experiment out of 2 experiments performed is shown. All data is represented as a distribution with the black line indicating the median

HSD is known to have a profound impact on the composition of the microbiota. Previous reports have shown that high salt diet feeding in mice resulted in depletion of *Lactobacillus* and enrichment of *Bifidobacterium,* which contributed to the altered T_H_17 and NK cell responses [10, 17]. Similar to published studies, while studying HSD-induced changes in mouse models of autoimmunity, we observed significant changes in the gut microbial diversity in HSD-fed mice. The alpha diversity (Shannon diversity index) was not different under HSD—indicating that there were no significant alterations in the overall diversity or species richness of the microbiota (Supplementary Fig. 2a). However, we found several OTUs were differentially prevalent under C1000 or HSD and principal coordinate analysis showed distinct clustering of samples according to diet (Supplementary Fig. 2b, c). Notably, we observed alterations in the relative abundance of several genera including Lactobacillus, Blautia, Enterococci, and Alistepes (Supplementary Fig. 2d). We subsequently analyzed this dataset for the presence of microbiota that has been previously associated with cancer. Enterococci have previously been reported to enhance immunotherapy responses [18, 19]*.* Interestingly, we found that HSD-fed mice had an increased abundance of Enterococci in their intestine (Supplementary Fig. 2e). We confirmed the increased abundance of Enterococci in HSD-fed C57BL/6 mice by qPCR (Fig. 1a). We also confirmed the ability of Enterococci to grow in high salt conditions by monitoring their growth in brain heart infusion (BHI) broth with or without 4% NaCl (Supplementary Fig. 2f). Both high sodium levels in the intestine and an altered microbiota may alter the intestinal barrier permeability, which may affect the migration of intestinal immune cells to peripheral organs where they can contribute to the local immune responses. We thus measured the permeability of the intestinal barrier using FITC-Dextran as a tracer. We did not find changes in the permeability of orally gavaged FITC-Dextran into blood, indicating there were no significant alterations to the barrier in HSD-fed mice (Fig. 1b). We affirmed this by quantification of albumin levels in the feces, which showed no significant leakage of serum albumin into the feces (Fig. 1c). We further quantified fecal IgA, as intestinal IgA production is influenced by the gut microbiota and altered IgA levels can influence immune homeostasis. Fecal IgA levels quantified through ELISA also showed no significant difference (Fig. 1d). From these data, we conclude that HSD alters gut microbiota with enrichment of cancer-associated Enterococci without altering intestinal permeability.

### HSD-induced alterations in immune cell populations

Subsequently, to understand the alterations in immunological responses under HSD, we performed immunophenotyping of the gut and peripheral immune compartments. Flow cytometry analysis in the spleen and small intestinal lamina propria (siLP) showed no significant changes in the frequencies of CD4^+^ T cells, CD8^+^ T cells, monocytes, neutrophils, and B cells in the spleen and siLP (Fig. 1f). The immune cells profile of the lymph nodes was also similar to that of the spleen, with no significant changes in the frequencies of CD4^+^ T cells, CD8^+^ T cells, monocytes, neutrophils, and B cells between C1000 and HSD fed mice (Supplementary Fig. 3a). Next, we analyzed the T cell compartment in detail to identify differences in various T cell subsets. We observed a significant decrease in IFN*γ*^+^ TNF*α*^+^ T cells in the spleen of HSD fed mice and the frequencies of other cytokine-producing cells were comparable between C1000 and HSD fed mice in both spleen and lymph nodes (Fig. 1g, Supplementary Fig. 3b). In line with previous findings [20, 21], we also noted an elevated frequency of IL-17a producing CD4^+^ T cells in the small intestine lamina propria in HSD-fed mice, whereas the frequencies of other cytokines producing (IFN*γ*, TNF*α*) T cells remained similar (Fig. 1h, Supplementary Fig. 3d–f). Further, given that targeting PD1 has been an effective immunotherapeutic intervention in cancer and that HSD had been shown previously to affect anti-PD-1 therapy [10], we compared the frequencies of PD1^+^ T cells and found no significant differences in HSD-fed mice (Fig. 1f). Since NK cells are an essential part of tumor immunosurveillance, we analyzed the NK cell compartment and found that their frequency was marginally reduced in the spleen of HSD-fed mice (Fig. 1e). The splenic NK cells also showed a lower expression of perforin, without any change in the granzyme expression (Fig. 1i-j, Supplementary Fig. 3c).

It is also now well established that Foxp3^+^ T_reg_ cells have a significant role in several types of cancers, and targeting them has been shown to significantly enhance anti-tumor immune responses [22]. Hence, we analyzed the Foxp3^+^ T_reg_ cell population in the colonic lamina propria and found that their frequency was elevated in the intestine of HSD-fed mice (Fig. 1k). Interestingly, though their frequencies were not different in the periphery, the isolation and culture of lymph node T_reg_ cells showed that they produced more IL-10 under HSD (Fig. 1l). Together, these data showed that HSD modulates the immune cell effector functions, especially NK cells and CD4^+^ T cell subsets.

### HSD does not alter AML progression

**Fig. 2:**
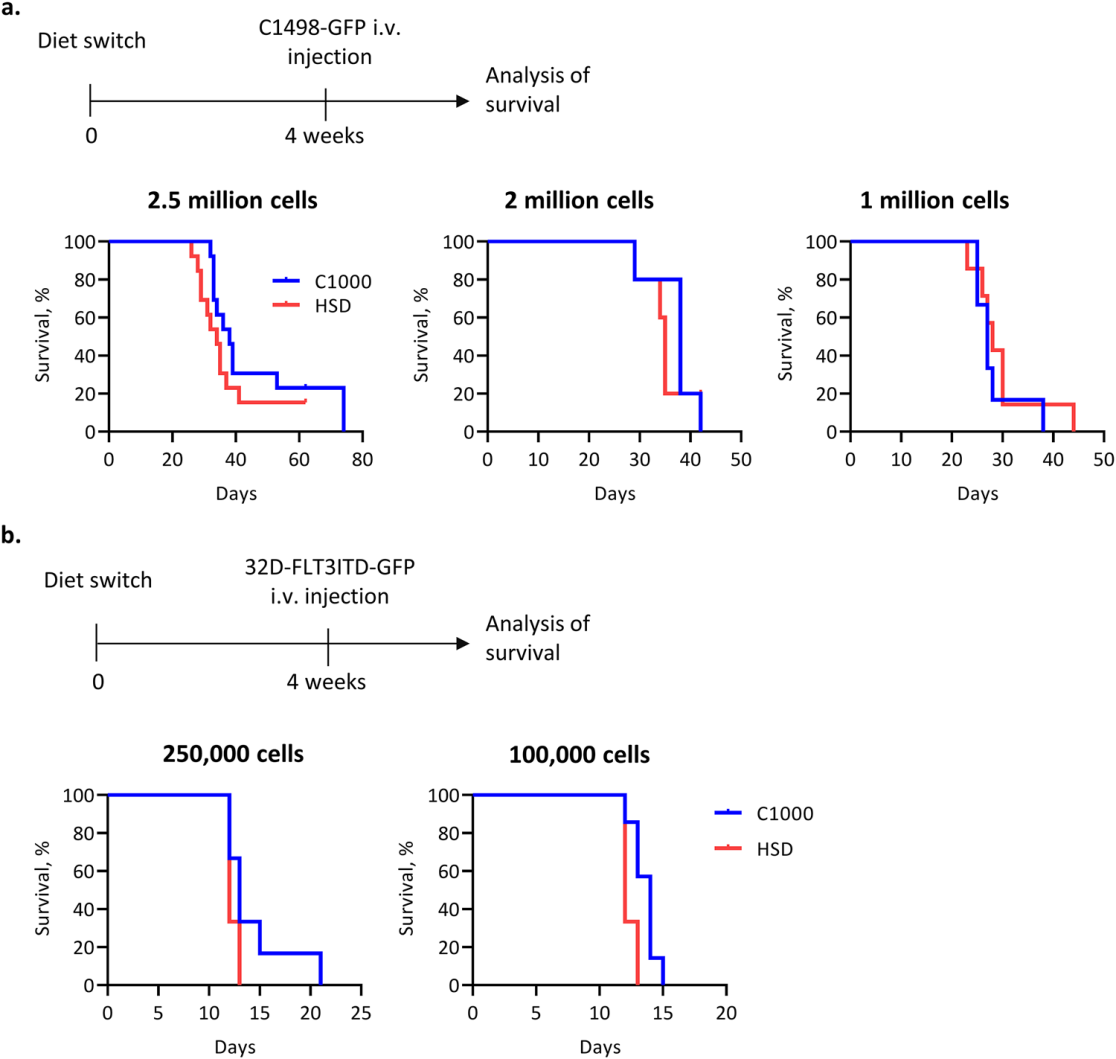
HSD does not alter AML progression. **a** Experimental plan (top) and survival plots (bottom) for AML progression in C57BL/6 mice fed C1000 or HSD. Mice were fed the respective diets for 4 weeks after which different numbers of C1498-GFP cells were injected i.v. *n* = 5–13 mice per group. **b** Experimental plan (top) and survival plots (bottom) for AML progression in WT C3H/HeJ mice fed C1000 or HSD. Mice were fed the respective diets for 4 weeks after which different numbers of 32D-FLT3ITD-GFP cells were injected i.v. *n* = 6–7 mice per group

To assess the effects of HSD on hematologic cancer, we used 2 different syngeneic transplantation models. The injection of C1498 cells, a murine AML cell line isolated from C57BL/6 mice, leads to the invasion and aggressive growth within the bone marrow, spleen, liver, lung, and ovaries [23]. The second model utilizes a myeloblast-like cell line 32D cells from C3H/HeJ mice transduced with FMS-like tyrosine kinase 3 (FLT3) with internal tandem duplications (FLT3ITD), a constitutively active receptor tyrosine kinase found in 25–30% of AML patients [24]. After injection of cells into immunocompetent syngeneic mice, aggressive acute leukemia is observed after an asymptomatic prodromal period of 10–20 days. We fed the mice with HSD or control diet 4 weeks before the inoculation of tumor cells to reliably modify the microbiome and immune parameters (Fig. 2a, b). In both models of AML, HSD had no significant impact on disease development. We found aggressive growth of tumor cells in both groups of mice resulting in death (Fig. 2a, b). We also lowered the tumor cell inoculum to observe even marginal differences in disease development. However, we failed to find any significant differences between HSD and control diet fed mice (Fig. 2a, b).

## Discussion

Despite the constant advances in the development of treatment options for AML, there is an unmet need to understand new ways to modulate AML progression. In recent years, the gut microbiota has emerged as a novel player that can modulate the efficacy of cancer therapeutics in some epithelial cancers and melanoma in mice as well as in patients [19, 25–30]. Indeed, the presence of certain gut microbial species such as Enterococci has been strongly associated with treatment responses [19, 25–30]. Since the microbiota is amenable to rapid changes through diet or other lifestyle factors, the microbiota is considered an attractive therapeutic target in cancers. However, there is limited data available regarding the relevance of microbiota and the relevant microbial species that can be targeted for therapeutic interventions. Moreover, dietary interventions have been considered a natural way to modulate microbiota composition.

Recently, HSD has attracted attention owing to its effect on immune responses and the susceptibility to certain autoimmune diseases like colitis and Multiple Sclerosis [17, 31]. This has now been extended to certain types of cancers in mouse models. Although systemic immune responses are also affected in HSD-fed mice, the majority of the HSD-mediated functional changes were noted at the barrier sites such as the skin and intestine [16, 31]. Hence it is logical that tumors developing on the skin such as melanoma are affected by the HSD [10–12]. However, currently, it is unknown whether microbiota and HSD would play a role in blood tumors such as AML.

Previous studies have reported that HSD feeding in mice resulted in dysbiosis of the gut microbiota with an increased abundance of *Bifidobacterium* [10] and a decrease in *Lactobacillus* species [17]. Consistent with the published reports, we found that HSD-fed mice had altered microbiota composition including changes in the relative abundance of several genera including *Lactobacillus, Blautia, Enterococci, and Alistepes* and the cancer-associated *Enterococcus hirae*. It is also important to consider that the influence of diet on microbiota is dependent on the persistent exposure to the HSD and the changes in the microbiota are short-lived if the dietary exposure has been removed [32]. Indeed, we observed that the increased abundance of *Enterococcus hirae* required continued exposure to HSD. Hence, we fed our mice with HSD throughout the experiment to reliably modify the microbiome and immune cell profiles.

Besides their influence on the gut microbiota, HSD has a significant impact on immune responses, especially within the gut microenvironment. The notable immunological changes that were previously reported in the literature were induction of TH17 cells [17, 20, 21, 31], changes in NK cell functions [10], and inhibition of PD-1 expression (10). We confirmed that HSD-fed mice had increased frequencies of TH17 cells in their intestine and found that HSD treatment reduced the frequencies of NK cells and their perforin expression. While some reports found HSD enhances NK cell functions, others found HSD impairs NK cell functions [10, 33]. PD-1 expression in T cells was not different between C1000 and HSD-fed mice in our study. Interestingly, we found increased production of IL-10 production by Foxp3 cells from HSD-fed mice. While the precise contribution of each of these immune cell populations to AML development and progression is unclear, our experience in the mouse models used in this study suggests that NK cells and T cells contribute to the progression of AML.

Despite their profound effects on the microbiota and immune cells, we found no significant influence of HSD on AML development and progression. The kinetics and characteristics of the tumor were similar despite varying the number of injected tumor cells. The lack of impact of HSD on tumor development observed in our study is different from previously published studies in mouse models of melanoma and mammary cancer [10–12]. To our knowledge, this is the first study that evaluated the role of HSD in an AML model. One potential reason for the lack of effect of HSD observed here could be our choice of the relatively aggressive AML models used in this study. Despite the inherent limitations and their inability to faithfully recapitulate the complex biology of AML, various mouse models of AML have been useful in studying some selected biological aspects of AML biology [34]. One of the models used in the study uses a cell line that harbors a commonly observed mutation in AML (FLT3-ITD). However, the transfer of large numbers of transformed cells results in a relatively short and aggressive disease course that may not fully represent human disease. The mutation in a large population of genetically identical cells also fails to appropriately model clonal selection from a single cell. It is possible that the time window for HSD to influence tumor-mediated immune responses is not sufficient in this model system. Hence, it is essential to verify the impact of HSD on other models where the disease development is slower and less aggressive. Further, HSD-induced changes in the microbiota and the immune system can vary depending on the initial microbial composition of the gut and may lead to different effects of HSD across different colonies. Moreover, the microbiota has high genetic and functional redundancy to be resilient to various perturbations, therefore changes in microbial composition need not always have a functional effect.

While it is clear that the microbiota is one of the major influencers of many physiological and pathological responses in several diseases including cancer, our study shows that the effects of the microbiome and dietary interventions can be tumor-specific and may not apply to all types of cancer. Moreover, the microbiota composition is exceedingly diverse in humans due to their vast differences in lifestyle and environment. In addition, there are several subtypes of AML in humans, and the microbiota and immune alterations may not be similar in all of them. More research is needed to further understand the distinct tumor-specific microbiome and dietary interventions.

## Materials and methods

### Mice

Wild-type (WT) C57BL/6, C3H/HeJ mice, and OSE mice [35] were bred and housed at the animal facilities of the Max Planck Institute of Biochemistry, Martinsried. Mating pairs were fed a purified control diet (C1000) or High Salt Diet (HSD) containing 4% NaCl for the indicated periods. The diets were given ad libitum. Mice were given autoclaved drinking water ad libitum. The diets were formulated with Altromin and *γ*-irradiated. In diet switched mice, body weight measurements were done once every week from the day of the diet switch until 4 weeks after the diet switch. All animal procedures were performed following the guidelines of the Committee on Animals of the Max Planck Institute of Biochemistry and with approval from the Regierung von Oberbayern (Munich, Germany).

### AML induction

WT C57BL/6 and C3H/HeJ mice were randomly switched to either C1000 and HSD and maintained on the diets for 4 weeks, after which the C1498-GFP cells (for C57BL/6) [23] or the 32D-FLT3ITD-GFP cells [24] (for C3H/HeJ) were injected through i.v. via the tail vein. The disease was monitored and survival was analyzed up to 80 days after injection. Mice were maintained on their respective diets throughout the experiment.

### Blood collection

Unless otherwise specified, all blood collections were done after 3–4 weeks of feeding. For serum collection, blood was collected by retro-orbital bleeding into Serum gel tubes (Sarstedt) and allowed to stand at RT for 1 h. They were then centrifuged (10,000 rpm, 5 min, 4 °C) to collect serum. For complete blood count analysis, blood was collected onto EDTA tubes and analyzed using the Procyte Dx analyzer (IDEXX Hematology resources).

### Lymphocyte isolation

Single-cell suspensions were prepared from spleen or lymph nodes by mechanical disruption via forcing through 40 µm cell strainers. Cells were collected in RPMI (RPMI 1640, Sigma Aldrich) containing 10% heat-inactivated Fetal Bovine Serum (FBS, Sigma Aldrich). Spleen cells were further resuspended in erythrocyte lysis buffer (0.83% NH_4_Cl) and incubated for 3 min at room temperature. The lysis buffer was then neutralized with RPMI containing 10% FBS and the cells were washed and collected in FACS buffer for staining.

Small intestine and colon were collected in ice-cold HBSS (Thermo scientific) containing 15 mM HEPES (Sigma Aldrich). After careful removal of Peyer’s Patches, fatty tissue, and fecal content, the intestine was opened longitudinally and cut into small pieces. The intestinal fragments were washed three times for 15 min each, with magnetic stirring (300 rpm) in HBSS containing 5% FBS, 15 mM HEPES, and 5 mM EDTA. Next, intestinal pieces were washed for 5 min in RPMI containing 5% FBS and 15 mM HEPES, followed by enzymatic digestion for 1 h (37 °C, 550 rpm stirring speed) with Collagenase VII (Sigma Aldrich) diluted to 100 U/ml in RPMI containing 5% FBS and 15 mM HEPES. Digested tissue was forced through 100 µm cell strainers and washed twice in HBSS containing 5% FBS, 15 mM HEPES, and 5 mM EDTA. The pellet was resuspended in 5 ml of 40% Percoll (GE Healthcare Life Sciences), overlaid on 2.5 ml of 80% Percoll and centrifuged (2000 g, 20 min, room temperature, brakes set to 4). Cells at the interface were collected, washed, and resuspended in RPMI containing 10% FBS for culture, or in FACS buffer for staining.

### Flow cytometry

In samples where cytokine expression was not analyzed, cells were directly stained after isolation. In samples that were analyzed for cytokine expression, cells were activated with 50 ng/ml PMA and 500 ng/ml ionomycin in the presence of 5 µg/ml brefeldin A for 4 h at 37 °C before staining. For detection of cell surface markers, cells were washed twice with FACS buffer (PBS containing 1% bovine serum albumin (BSA; Carl-Roth) and 0.1% NaN_3_) and stained with the following antibodies used at concentrations between 1:100 and 1:400: anti-CD4 (RM4-5), anti-CD45 (30-F11), anti-CD19 (1D3), anti- CD11b (M1/70), anti- Ly6G (1A8), anti-Ly6C (AL-21), anti-NK1.1 (PK136), anti-PD1 (J43), anti-CD25 (PC61), anti-CD8 (53-6.7). Fixable viability dye eFluor-780 (Thermo Fisher Scientific) was used at a 1:1000 concentration. Cells were then washed twice in FACS buffer and either resuspended in FACS buffer for acquisition or used for intracellular staining. If cells were to be sorted, FACS buffer was replaced by PBS containing 1% BSA.

For intracellular/intranuclear staining, surface-stained cells were fixed and permeabilized by incubation with 100 µl of Fixation/Permeabilization Buffer (Transcription factor staining set, eBioscience). Cells were then stained with the following antibodies used at concentrations between 1:100 and 1:200: anti- IFN*γ* (XMG1.2), anti-IL-17A (TC11-18H10.1), anti- TNF*α* (MP6-XT22), anti-Perforin (S16009A), anti-Granzyme (QA16A02), anti- FoxP3 (FJK-16s). Finally, cells were washed twice in Permeabilization Buffer and resuspended in FACS buffer for acquisition.

Antibodies were purchased from BD Biosciences, Biolegend, or Pharmingen. Antibodies were used in conjugation with one of the following fluorophores: FITC, PE, PerCP-Cy5.5, PeCy7, APC, APC-Cy7, BV421, eFluor450, eFluor780, BV605, BV711, BV784, or APC-R700. Stained samples were acquired on FACS Canto (BD Biosciences), FACS Fortessa (BD Biosciences), or FACS Aria III (BD Biosciences). For sorting, FACS Aria III was used. Analysis was performed using FlowJo (TreeStar) software.

### Ex vivo* cell culture*

96 well flat-bottomed plates were precoated with 2 µg/ml of anti-CD3 (BioXCell) and 2 µg/ml of anti-CD28 (BioXCell) for 3 h at 37 °C. Sorted CD25^+^ cells from the cervical lymph node were then plated in these plates (2 × 10^5^ cells/well) and incubated at 37 °C for 60 h after which culture supernatants were collected and frozen at − 20 °C for ELISA.

### Intestinal barrier permeability assay

WT C57BL/6 mice fed C1000 or HSD for 3–4 weeks were withdrawn from food and water for 4 h, after which FITC-dextran 4 kDa (Sigma Aldrich) was orally gavaged to the mice at a concentration of 10 mg in 400 µl of PBS per mouse. Mice were further kept withdrawn from food and water for 4 h after which blood was collected and centrifuged (10,000 rpm, 5 min) to collect serum. The fluorescence of FITC in the serum was measured in 96 well black half area plates (Perkin Elmer) at an excitation of 485 nm and emission of 515 nm. FITC-dextran 4 kDa diluted in PBS was used as the standard.

### ELISA

ELISAs for IL-10, fecal albumin, and serum leptin were performed using the following kits according to the manufacturer’s instructions: Mouse IL-10 ELISA MAX Standard (Biolegend), Mouse Albumin ELISA quantitation kit (Bethyl laboratories), Mouse Leptin ELISA kit (ENZO life sciences).

For fecal IgA measurement, 96 well maxisorp plates (Thermo Scientific) were coated overnight at 4 °C with anti-IgA (C10-3, Pharmingen) in PBS. Plates were then washed with ELISA wash buffer (PBS containing 0.05% Tween-20 (Sigma Aldrich)) and blocked with PBS containing 10% FBS for 1 h. IgA standard (M18-254, Pharmingen) and fecal supernatants (diluted 1:10) were added and incubated for 2 h. Subsequently, plates were washed and incubated with biotin-labeled IgA primary antibody (C10-1, Pharmingen) for 1 h. Plates were washed and Streptavidin-HRP (Biolegend) was added. Lastly, TMB substrate solution (eBioscience) was added and the reaction was stopped with 1 N H_2_SO_4_. Plates were read at 450 nm.

### Fecal DNA extraction and qPCR

Fecal pellets and cecal contents were collected 3–4 weeks after the diet switch and frozen at − 80 °C until further use. Samples were thawed to RT. Then glass beads were added along with 500 µl of ASL buffer (Qiagen stool DNA kit). Pellets were homogenized using Tissuelyzer at 2 Hz for 7 min. They were incubated at 95 °C for 5 min and then homogenized again using Tissuelyzer at 25 Hz for 7 min. They were then centrifuged (13,000 rpm, 1 min, 4 °C). The pellet was mixed with 20 mg/ml of Lysozyme (Sigma Aldrich) in ASL buffer and incubated at 37 °C for 30 min. It was then homogenized using Tissuelyzer at 25 Hz for 7 min, incubated at 95 °C for 5 min, then homogenized again using Tissuelyzer at 25 Hz for 7 min, and then centrifuged (13,000 rpm, 1 min, 4 °C). The supernatant was collected and subsequent steps were done with a Qiagen stool DNA kit according to the manufacturer’s instructions.

For qPCR, isolated DNA was diluted to 2 ng/µl. qPCR was performed with an Absolute qPCR master (Thermo scientific) mix using SYBR green probe. Separate PCR reactions were performed using 16S rRNA universal primers (ACTCGTTGTACTTCCCATTGT; CCCTTATTGTTAGTTGCCATCATT) and Enterococci-specific 16S rRNA primers (ATTACCGCGGCTGCTGGC; ACTCCTACGGGAGGCAGCAGT). The Enterococci 16S rRNA gene encoded in a linearized plasmid was used as the DNA standard for both PCRs. Total fecal bacterial levels were computed using the standard curve obtained from universal primers and Enterococci levels were computed using the standard curve obtained from Enterococci-specific primers. Enterococci levels were then normalized to total bacterial levels.

### DNA extraction and sequencing

DNA was extracted from caecal content using the NucleoSpin DNA stool mini kit (Macharey Nagel). For microbiota sequencing, DNA was diluted to 1 ng/μL. The DNA samples were processed and sequenced by Novogene. Briefly, 16S rRNA genes were amplified using the Phusion® High-Fidelity PCR Master Mix (New England Biolabs) with the 16S V3-V4 specific primers. PCR products were run on a 2% agarose gel for detection. Samples with a bright main strip between 400 and 450 bp were chosen for further experiments. PCR products were mixed at equal density ratios and purified with the Qiagen Gel Extraction Kit. Libraries were generated with NEBNext® Ultra™ DNA Library Prep Kit (New England Biolabs) and sequenced by the Illumina platform. Paired-end reads were merged using FLASH and chimeric sequences were removed using the UCHIME algorithm. Sequence analysis was performed using Uprase. Sequences having ≥ 97% similarity were assigned to the same OTU. Representative sequences for each OTU were screened for further annotation. The SILVA rRNA database was used for species annotation at each taxonomic rank. The phylogenetic relationship between OTUs was obtained using MUSCLE (Version 3.8.31). OTU abundance information was normalized using a standard sequence number corresponding to the sample with the least sequences. Subsequent analyses of alpha diversity and beta diversity were all performed based on this output normalized data. Diversity analyses were performed using the R packages MicrobiotaProcess and Phyloseq. Shannon index was used to compute the alpha diversity. A principal coordinate analysis (PCoA) plot with Bray–Curtis dissimilarity was used for beta diversity. The clean sequencing reads were submitted to NCBI Short Read Archive under BioProject identifier PRJNA825562.

### Statistical analysis

GraphPad Prism 9 (GraphPad Software, Inc.) was used for all statistical analyses. Information on statistical tests used for analysis is mentioned in figure legends. P values below 0.05 were considered significant.

### Supplementary Information

Below is the link to the electronic supplementary material.

**Supplementary figure 1: HSD is well tolerated by mice.** (**a**) The chart shows the relative proportions of carbohydrates, minerals, sodium, chloride, and moisture in C1000 and HSD diets. (**b**) Bodyweight change in C57BL/6 mice after diet switch (C1000 *n* = 7, HSD *n* = 7). Data represented as mean ± s.e.m. (**c**) Leptin levels in WT mice 4 weeks after diet switch (C1000 n=19, HSD *n* = 17). Data from sera collected across 3 cohorts of mice is shown. Each circle represents an individual mouse. Data is represented as a distribution with the black line indicating the median (**d**) Heatmap of the complete blood count analysis on blood collected from C1000 (*n* = 16) and HSD (*n* = 16) fed C57BL/6 mice. *P<0.05, ** *P*<0.01, ***P<0.001 (Mann Whitney’s *U* test). Values for all parameters were z-scored across the rows and represented in the heatmap. Data from sera collected across 2 cohorts of mice is shown. RBC, red blood cells; MCV, mean cell volume; RDW-SD, red blood cell distribution width-Standard deviation; PDW, platelet distribution width; MPV, mean platelet volume; MCHC, mean corpuscular hemoglobin concentration; HGB, hemoglobin; RET-He, reticulocyte Hb equivalent; RDW-CV, red blood cell distribution width-Coefficient of variation; IRF, immature reticulocyte fraction; HCT, hematocrit; RET, reticulocyte; LFR, reticulocytes with low fluorescence; MFR, reticulocytes with medium fluorescence; HFR, reticulocytes with high fluorescence; P-LCR, platelet-large cell ratio; PCT, procalcitonin; NEUT, neutrophils; LYMPH, lymphocytes; MONO, monocytes; EO, eosinophils; BASO, basophils; RET, reticulocytes; PLT, platelets; WBC, white blood cells; M/ul, millions per cubic milliliter; fl, femtoliters; g/dl, grams per deciliter; pg, picogram; K/ul thousands per cubic milliliter. **Supplementary figure 2: Gut microbiota alterations induced by HSD.** (**a**–**e**) OSE mice were weaned onto C1000 (*n* = 8) or HSD (*n* = 8) and caecal content was collected 3-4 weeks after the diet switch. DNA was extracted and the 16s rRNA gene was sequenced. (**a**) Shannon diversity index of caecal microbiota from C1000 and HSD-fed mice. Each circle represents an individual mouse. Data is represented as a distribution with the black line indicating the median. (**b**) Venn diagram indicating the prevalence of the identified microbial OTUs in C1000 and HSD fed mice. (**c**) Principal coordinate analysis using Bray-Curtis dissimilarity matrix. (**d**) Heatmap showing the relative abundance of the most differentially abundant genera under C1000 and HSD (computed as a percentage of the total, then z-scored and represented). (**e**) 16s rRNA qPCR analysis of fecal DNA with Enterococci specific primers represented as a percentage of total microbiota (C1000 *n* = 4, HSD *n* = 5). OSE mice were weaned onto C1000 or HSD and fecal content was collected 3–4 weeks after the diet switch; *P = 0.015 (Mann Whitney’s *U* test). Each circle represents an individual mouse. One representative experiment out of 2 experiments performed is shown. Data is represented as a distribution with the black line indicating the median. (**f**) OD (600nm) at various time points during the culture of *Enterococcus hirae* and *Escherichia coli* in Brain Heart Infusion Broth (BHI) broth with or without 4 % NaCl. Data represented as mean ± s.e.m. **Supplementary figure 3: HSD alters cytokine-producing T cell populations.** (**a**–**f**) C57BL/6 mice were weaned onto C1000 or HSD and lymph nodes were collected 3–4 weeks after the diet switch. (**a**) Frequencies (% of CD45^+^ cells) of various immune cell subsets in the lymph nodes of C57BL/6 mice fed with C1000 and HSD. *n* = 9–11 per group. Data from 3 individual experiments are pooled. (**b**) Frequencies (% of CD4^+^T cells) of various cytokine-producing T cell subsets in the lymph nodes of C57BL/6 mice fed with C1000 and HSD. Data from 3 individual experiments are pooled. *n* = 9–11 per group. (**c**) Representative flow cytometry histograms showing the MFI of Perforins in splenic NK cells from C1000 and HSD fed mice. (**d**) Representative flow cytometry plots showing TNFα and IFNγ staining in splenic CD4^+^T cells from C1000 and HSD-fed mice. (**e**) Representative flow cytometry plots showing IL17 staining in SILP CD4^+^T cells from C1000 and HSD fed mice. (**f**) Representative flow cytometry plots showing Foxp3 staining in colonic CD4+T cells from C1000 and HSD fed mice. (PDF 406 KB)
