# Supplementary material for: High salt diet does not impact the development of acute myeloid leukemia in mice
Source: Cancer Immunol Immunother. 2022 Jul 8;72(1):265–73. doi: 10.1007/s00262-022-03244-y (PMC9813099; doi:10.1007/s00262-022-03244-y)
Supplement: Supplementary file 1 — Supplementary figure 1: HSD is well tolerated by mice. (a) The chart shows the relative proportions of carbohydrates, minerals, sodium, chloride, and moisture in C1000 and HSD diets. (b) Bodyweight change in C57BL/6 mice after diet switch (C1000 n = 7, HSD n = 7). Data represented as mean ± s.e.m. (c) Leptin levels in WT mice 4 weeks after diet switch (C1000 n=19, HSD n = 17). Data from sera collected across 3 cohorts of mice is shown. Each circle represents an individual mouse. Data is represented as a distribution with the black line indicating the median (d) Heatmap of the complete blood count analysis on blood collected from C1000 (n = 16) and HSD (n = 16) fed C57BL/6 mice. *P<0.05, ** P<0.01, ***P<0.001 (Mann Whitney’s U test). Values for all parameters were z-scored across the rows and represented in the heatmap. Data from sera collected across 2 cohorts of mice is shown. RBC, red blood cells; MCV, mean cell volume; RDW-SD, red blood cell distribution width-Standard deviation; PDW, platelet distribution width; MPV, mean platelet volume; MCHC, mean corpuscular hemoglobin concentration; HGB, hemoglobin; RET-He, reticulocyte Hb equivalent; RDW-CV, red blood cell distribution width-Coefficient of variation; IRF, immature reticulocyte fraction; HCT, hematocrit; RET, reticulocyte; LFR, reticulocytes with low fluorescence; MFR, reticulocytes with medium fluorescence; HFR, reticulocytes with high fluorescence; P-LCR, platelet-large cell ratio; PCT, procalcitonin; NEUT, neutrophils; LYMPH, lymphocytes; MONO, monocytes; EO, eosinophils; BASO, basophils; RET, reticulocytes; PLT, platelets; WBC, white blood cells; M/ul, millions per cubic milliliter; fl, femtoliters; g/dl, grams per deciliter; pg, picogram; K/ul thousands per cubic milliliter. Supplementary figure 2: Gut microbiota alterations induced by HSD. (a–e) OSE mice were weaned onto C1000 (n = 8) or HSD (n = 8) and caecal content was collected 3-4 weeks after the diet switch. DNA was extracted and the 16s rRNA gen [file 262_2022_3244_MOESM1_ESM.pdf]

Supplementary figures

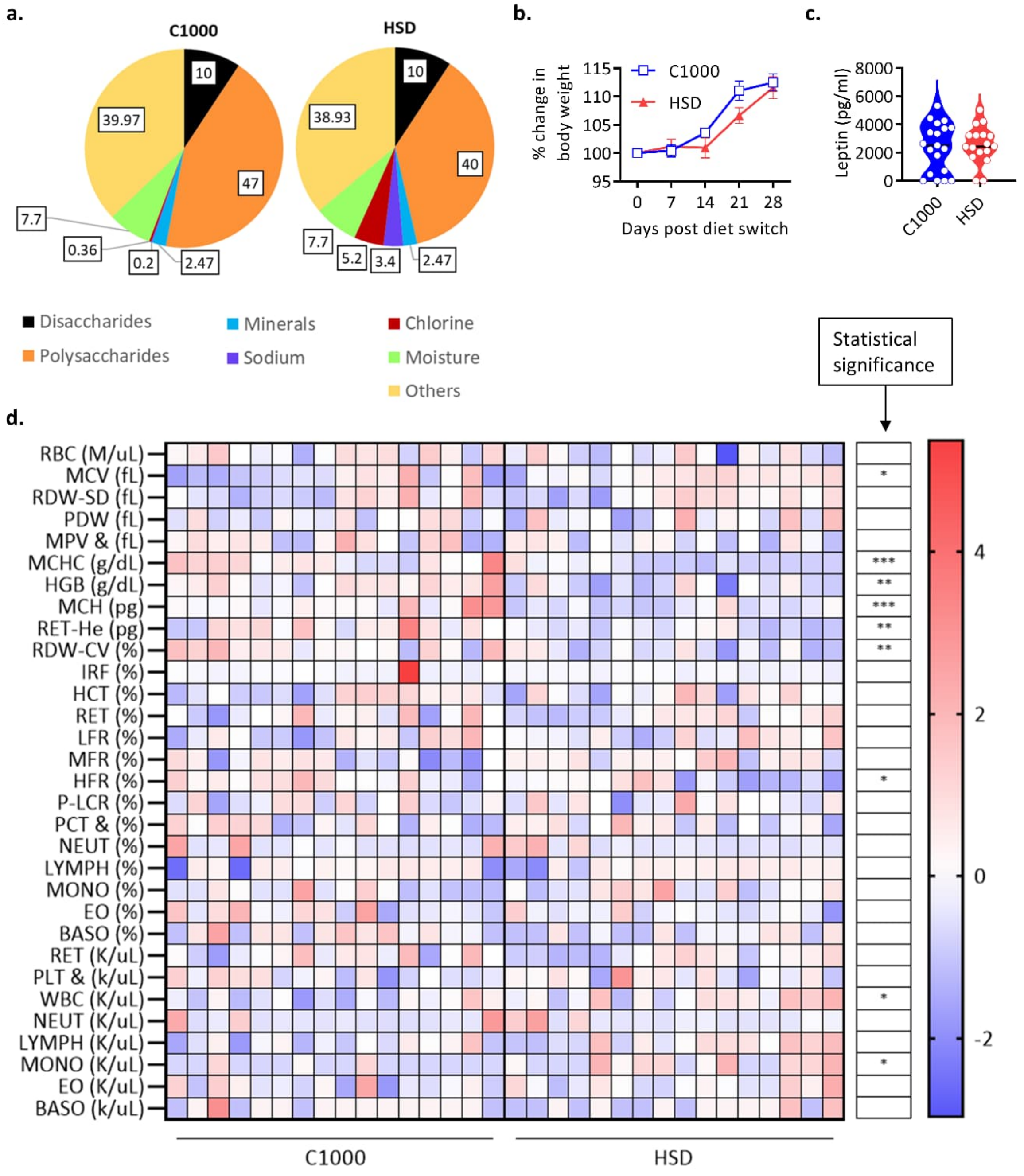

Supplementary figure 1: HSD is well tolerated by mice. (a) The chart shows the relative proportions of carbohydrates, minerals, sodium, chloride, and moisture in C1000 and HSD diets. **(b)** Bodyweight change in C57BL/6 mice after diet switch (C1000 n = 7, HSD n = 7). Data represented as mean ± s.e.m. **(c)** Leptin levels in WT mice 4 weeks after diet switch (C1000 n=19, HSD n=17). Data from sera collected across 3 cohorts of mice is shown. Each circle represents an individual mouse. Data is represented as a distribution with the black line indicating the median.

(d) Heatmap of the complete blood count analysis on blood collected from C1000 (n = 16) and HSD (n = 16) fed C57BL/6 mice. *P<0.05, ** P<0.01, ***P<0.001 (Mann Whitney's U test). Values for all parameters were z-scored across the rows and represented in the heatmap. Data from sera collected across 2 cohorts of mice is shown. RBC, red blood cells; MCV, mean cell volume; RDW-SD, red blood cell distribution width-Standard deviation; PDW, platelet distribution width; MPV, mean platelet volume; MCHC, mean corpuscular hemoglobin concentration; HGB, hemoglobin; RET-He, reticulocyte Hb equivalent; RDW-CV, red blood cell distribution width-Coefficient of variation; IRF, immature reticulocyte fraction; HCT, hematocrit; RET, reticulocyte; LFR, reticulocytes with low fluorescence; MFR, reticulocytes with medium fluorescence; HFR, reticulocytes with high fluorescence; P-LCR, platelet-large cell ratio; PCT, procalcitonin; NEUT, neutrophils; LYMPH, lymphocytes; MONO, monocytes; EO, eosinophils; BASO, basophils; RET, reticulocytes; PLT, platelets; WBC, white blood cells; M/ul, millions per cubic milliliter; fl, femtoliters; g/dl, grams per deciliter; pg, picogram; K/ul thousands per cubic milliliter.

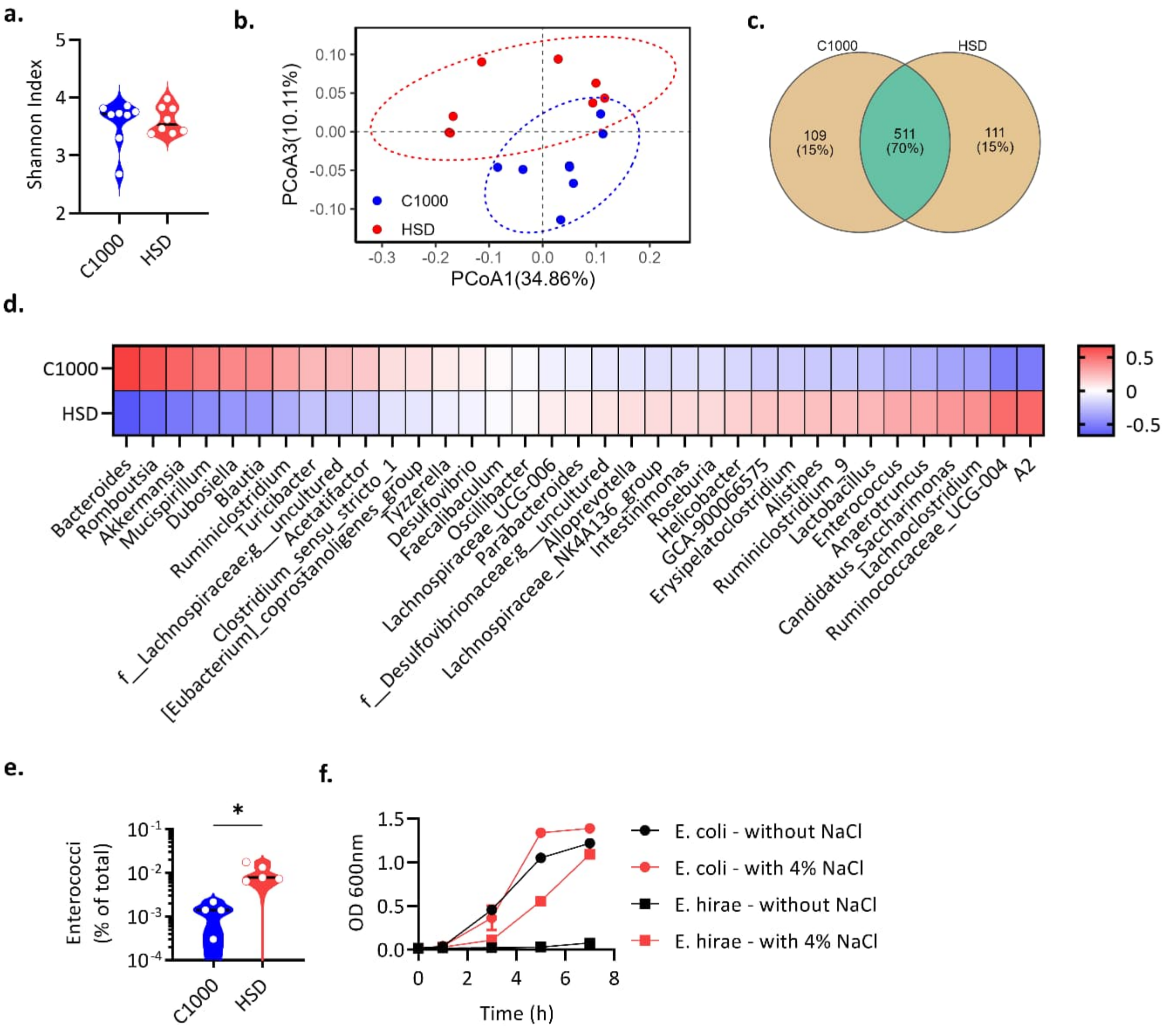

Supplementary figure 2: Gut microbiota alterations induced by HSD. (a-e) OSE mice were weaned onto C1000 (n = 8) or HSD (n = 8) and caecal content was collected 3-4 weeks after the diet switch. DNA was extracted and the 16s rRNA gene was sequenced. **(a)** Shannon diversity index of caecal microbiota from C1000 and HSD-fed mice. Each circle represents an individual mouse. Data is represented as a distribution with the black line indicating the median. **(b)** Venn diagram indicating the prevalence of the identified microbial OTUs in C1000 and HSD fed mice. **(c)** Principal coordinate analysis using Bray-Curtis dissimilarity matrix. **(d)** Heatmap showing the relative abundance of the most differentially abundant genera under C1000 and HSD (computed as a percentage of the total, then z-scored and represented). **(e)** 16s rRNA qPCR analysis of fecal DNA with Enterococci specific primers represented as a percentage of total microbiota (C1000 n = 4, HSD n = 5). OSE mice were weaned onto C1000 or HSD and fecal content was collected 3 – 4 weeks after the diet switch; *P = 0.015 (Mann Whitney's U test). Each circle represents an individual mouse. One representative experiment out of 2 experiments performed is shown. Data is represented as a distribution with the black line indicating the median. **(f)** OD (600nm) at various time points during the culture of *Enterococcus hirae* and *Escherichia coli* in Brain Heart Infusion Broth (BHI) broth with or without 4 % NaCl. Data represented as mean ± s.e.m.

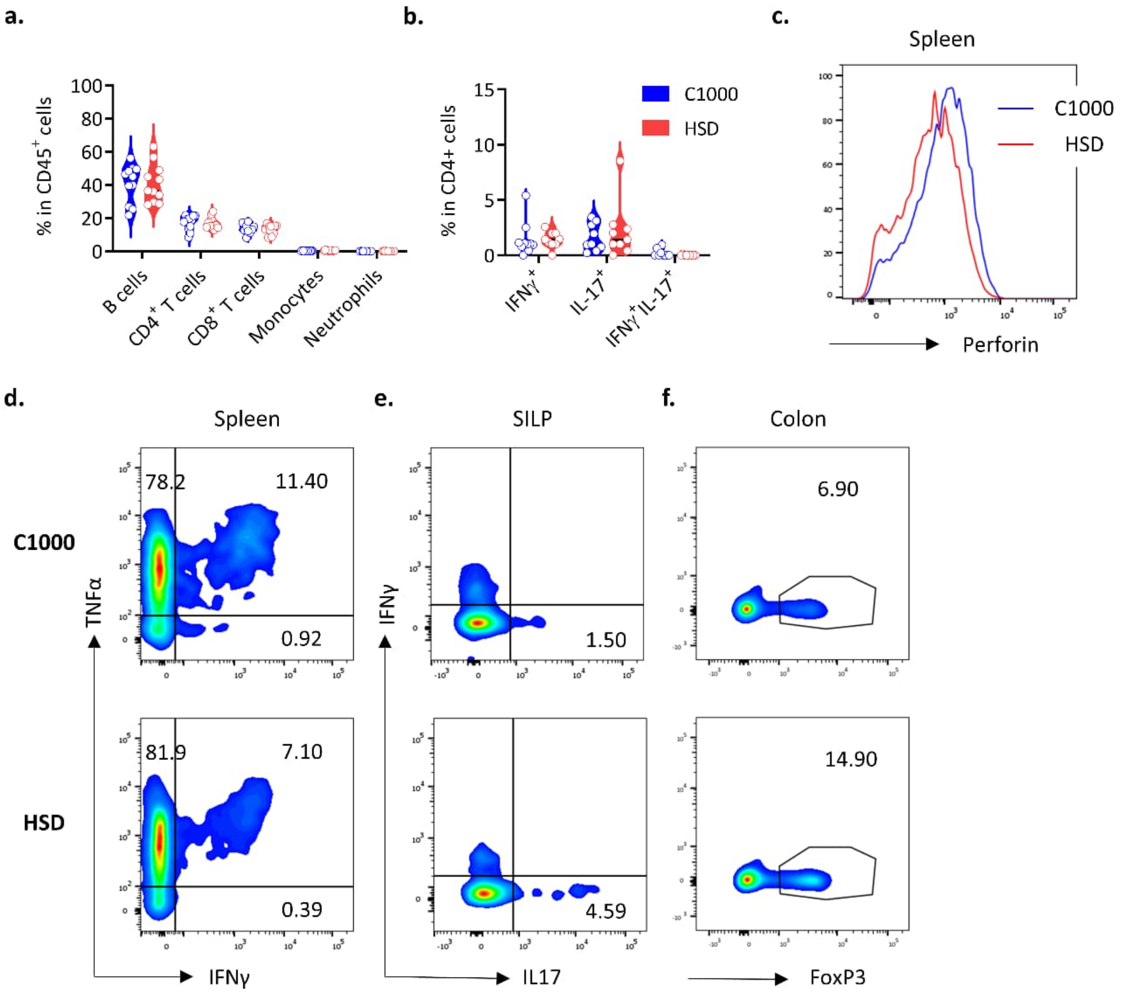

Supplementary figure 3: HSD alters cytokine-producing T cell populations. (a-f) C57BL/6 mice were weaned onto C1000 or HSD and lymph nodes were collected 3 – 4 weeks after the diet switch. **(a)** Frequencies (% of $CD45^+$ cells) of various immune cell subsets in the lymph nodes of C57BL/6 mice fed with C1000 and HSD. n = 9 – 11 per group. Data from 3 individual experiments are pooled. **(b)** Frequencies (% of $CD4^+$ T cells) of various cytokine-producing T cell subsets in the lymph nodes of C57BL/6 mice fed with C1000 and HSD. Data from 3 individual experiments are pooled. n = 9 – 11 per group. **(c)** Representative flow cytometry histograms showing the MFI of Perforins in splenic NK cells from C1000 and HSD fed mice. **(d)** Representative flow cytometry plots showing TNFα and IFNγ staining in splenic $CD4^+$ T cells from C1000 and HSD-fed mice. **(e)** Representative flow cytometry plots showing IL17 staining in SILP $CD4^+$ T cells from C1000 and HSD fed mice. **(f)** Representative flow cytometry plots showing Foxp3 staining in colonic CD4+ T cells from C1000 and HSD fed mice.
